# Suppression of Cytokine Release by Fluticasone Furoate vs. Mometasone Furoate in Human Nasal Tissue *Ex-Vivo*

**DOI:** 10.1371/journal.pone.0093754

**Published:** 2014-04-07

**Authors:** Nan Zhang, Koen Van Crombruggen, Gabriele Holtappels, Feng Lan, Michail Katotomichelakis, Luo Zhang, Petra Högger, Claus Bachert

**Affiliations:** 1 Upper Airway Research Laboratory, Ghent University Hospital, Ghent, Belgium; 2 Department of Otorhinolaryngology, Medical School, Democritus University of Thrace, Alexandroupolis, Greece; 3 Department of Otolaryngology Head and Neck Surgery, Beijing TongRen Hospital, Capital Medical University, Beijing, P.R.China; 4 Institute for Pharmacy and Food Chemistry, Julius-Maximilians-Universitity, Würzburg, Germany; 5 Division of ENT Diseases, Clintec, Karolinska Institutet, Stockholm, Sweden; Leiden University Medical Center, Netherlands

## Abstract

**Background:**

Topical glucocorticosteroids are the first line therapy for airway inflammation. Modern compounds with higher efficacy have been developed, but head-to-head comparison studies are sparse.

**Objective:**

To compare the activity of two intranasal glucocorticoids, fluticasone furoate (FF) and mometasone furoate (MF) with respect to the inhibition of T helper (Th)1, Th2 and Th17 cytokine release in airway mucosa.

**Methods:**

We used an ex-vivo human nasal mucosal tissue model and employed pre- and post- Staphylococcus aureus enterotoxin B (SEB)-challenge incubations with various time intervals and drug concentrations to mimic typical clinical situations of preventive or therapeutic use.

**Results:**

At a fixed concentration of 10^−10^ M, FF had significantly higher suppressive effects on interferon (IFN)-γ, interleukin (IL)-2 and IL-17 release, but not IL-5 or tumor necrosis factor (TNF)-α, vs. MF. While the maximal suppressive activity was maintained when FF was added before or after tissue stimulation, the cytokine suppression capacity of MF appeared to be compromised when SEB-induced cell activation preceded the addition of the drug. In a pre-challenge incubation setting with removal of excess drug concentrations, MF approached inhibition of IL-5 and TNF-α after 6 and 24 hours while FF maximally blocked the release of these cytokines right after pre-incubation. Furthermore, FF suppressed a wider range of T helper cytokines compared to MF.

**Conclusion:**

The study demonstrates the potential of our human mucosal model and shows marked differences in the ability to suppress the release of various cytokines in pre- and post-challenge settings between FF and MF mimicking typical clinical situations of preventive or therapeutic use.

## Introduction

Topical glucocorticosteroids (GCS) are the first line therapy for allergic rhinitis, sinusitis and nasal polyposis [1], [2]. Over the last decades, GCS compounds with higher efficacy and less systemic bioavailability have been developed, with fluticasone furoate (FF) as the latest compound being introduced to the market [3], [4]. FF has the molecular backbone of fluticasone that is also present in fluticasone propionate (FP) but the properties of both molecules are distinctly different [5]. Although FF has a higher receptor affinity and better tissue retention compared to mometasone furoate (MF) [6], [7], the clinical advantage of FF over MF has not been demonstrated yet in rigorous clinical trials. In absence of head-to-head comparison studies for both drugs, such comparison may also be extrapolated from ex-vivo human nasal mucosal models using diseased and control mucosa with different stimuli.

To optimally respond to the expectations of the patients, a nasal topical corticosteroid should have a fast onset of action, which requires a fast uptake and strong binding to the glucocorticoid receptor, translocation of the glucocorticoid receptor complex into the nucleus and interaction with glucocorticoid response elements (GREs), resulting in a long duration of action allowing for once daily application, and a high efficacy in suppressing the local inflammation with a broad panel of cytokines from Th1, Th2 and Th17 cells included [8].

We have developed an ex-vivo human mucosal tissue model for Staphylococcus aureus enterotoxin (SEB) induced T-effector cell activation [9], [10], in which we can interfere with glucocorticosteroids and other therapeutic compounds. The model is very robust and reproducible, but sensitive to demonstrate differences in the efficacy of compounds. At the same time, it is very close to the real-life situation and may thus predict the clinical outcome. We here used this model for a comparative study of FF vs MF in their efficacy, duration of their effect and onset of action. The inhibition of the release of a pattern of Th1, Th2 and Th17 cytokines was studied in three groups of experiments.

The experiments were designed to reflect optimal conditions for the drugs with incubation prior to and together with the stimulus, or real life conditions, with application of the drugs after initiation of disease exacerbation by a stimulus, and finally to study onset and duration of action after a defined exposure to the drug.

## Methods

### Human specimen

Nasal polyp samples were collected during functional endoscopic sinus surgery from 9 patients (median age, 52 years; range, 28-72 years; 5 men and 4 women). Nasal polyposis was diagnosed on the basis of symptoms, clinical examination, nasal endoscopy, and sinus computed tomography scan according to the European Position Paper on Rhinosinusitis and Nasal Polyps guidelines [1]. The atopic status of patients was evaluated by skin prick tests (SPTs) with the European standard panel of 14 inhalant allergens. Negative and positive controls (10 mg/mL histamine solution) were included with each SPT. 5 nasal polyp patients with positive SPT for at least 1 of the most common aeroallergens, 4 negative. Three patients reported mild asthma in their history, and one patient reported aspirin intolerance.

All patients were asked to refrain from topical or oral corticosteroids or antibiotics 4 weeks preoperatively and gave their written informed consent and the ethics committee of the Ghent University Hospital approved the study.

### Mechanical disruption of human nasal tissue

The nasal tissue was cut thoroughly as described before [9], [10] in tissue culture medium (TCM) of RPMI 1640 (Sigma-Aldrich, Bornem, Belgium) containing 2 mmol/L L-glutamine (Invitrogen, Merelbeke, Belgium), antibiotics (50 IU/mL penicillin and 50 mg/mL streptomycin; Invitrogen), and 0.1% BSA (Sigma). The tissue was passed through a mesh (+0.9 mm^2^) to achieve comparable fragments. The fragments (+0.9 mm^3^) were weighed, suspended as 0.04 g tissue/mL TCM and incubated for 1 hour at 37°C, 5% CO_2._ In a following step tissue fragments were washed, resuspended in the appropriate amount of TCM, and distributed into a 48-well plate (BD Falcon, VWR International, Belgium) with 0.5 mL tissue fragment suspension in each well.

### Pre-challenge incubation - the experiment to compare the effect of compounds under optimal conditions

The polyp tissue fragments were pre-incubated with TCM (RPMI+DMSO), 10^−8^ M, 10^−9^ M, 10^−10^ M and 10^−11^ M fluticasone furoate (FF; GSK Stevenage, UK) (29,6 mg FF dissolved in 5500 μl DMSO made a stock solution of 10 mM (10^−2^ M)) or mometasone furoate (MF; Schering Plough) (52,14 mg in 10 ml DMSO made a stock solution of 10 mM (10^−2^ M)) for 1 hour at 37°C/5% CO_2_ and then stimulated with TCM (negative control) or 0.5 μg/mL SEB (# S4881; Sigma) for 24 hours. The final concentration of DMSO was 0.1% in each well. Aliquots of the supernatants were stored immediately at −20°C until analysis of cytokines.

### Post challenge incubation - the experiment to compare the effect of drugs on a running stimulation

The polyp tissue fragments were stimulated with TCM (negative control) or 0.5 μg/mL SEB 1 hour or 2 hours or 4 hours then followed with TCM, 10^−8^ M, 10^−9^ M, 10^−10^ M and 10^−11^ M FF or MF for 24 hour at 37°C/5% CO_2_. Aliquots of the supernatants were stored immediately at −20°C until analysis of cytokines.

### Pre-challenge incubation with time interval - the experiment to compare the uptake and translocation of compounds

The polyp tissue fragments were pre-incubated with TCM, 10^−8^ M, 10^−9^ M, 10^−10^ M and 10^−11^ M FF or MF for 1 hour at 37°C/5% CO_2_, washed 2 times with TCM, and after 0 hour, 6 hours and 24 hours stimulated with TCM (negative control) or 0.5 μg/mL SEB for 24 hours. Aliquots of the supernatants were stored immediately at −20°C until analysis of cytokines.

### Measurements of T effector cell cytokines in supernatants of stimulated tissue fragments

Concentrations of the cytokines IFN-γ, IL-5 and IL-17, IL-1β, TNF-α, IL-2, IL-4, IL-8, IL-6, IL-10, IL-12p70, and IL-13 were measured in tissue supernatants obtained after the ex vivo stimulations using Multi-spot assays (Meso Scale Discovery, Gaithersburg, Maryland, USA) following the instructions of the manufacture. The plates were analyzed by using a Sector Imager 6000 (Meso Scale Discovery). Calculations of maximum percentage of inhibition for the different compounds, time points and cytokines were done.

### Statistical analysis

Statistical analysis was performed by using the Wilcoxon test (for paired comparisons). The Mann-Whitney U test was used for between-group (unpaired) comparisons. P values of less than .05 were considered statistically significant.

## Results

### Under optimal conditions, the suppressive effect of FF is superior to MF

**Figure 1:**
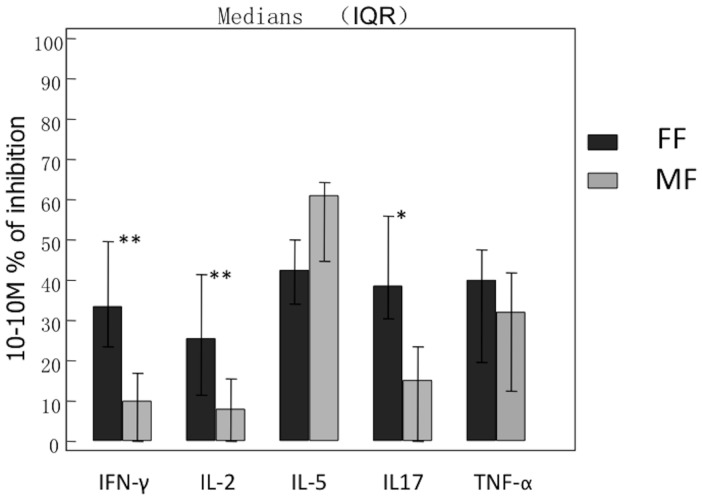
Nasal polyp tissue (n = 9) was pre-incubated with FF or MF at different concentrations for 1 hour and consequently stimulated with SEB for 24 hours (pre-challenge incubation). % inhibition of cytokine release was calculated at a fixed concentration of 10^−10^ M; FF had a significantly higher suppressive effect on IFN-γ, IL-2 and IL-17 release vs. MF.

FF and MF showed a dose-dependent suppressive effect on the release of IFN-γ, IL-2, IL-5, IL-17, and TNF-α upon stimulation. There were no significant differences in maximum percentage of inhibition of major cytokines between FF and MF after pre-challenge incubation conditions (Table 1). However, FF reached a significant suppressive effect at a 1-molar or lower concentration for all cytokines. We further calculated the percentage of inhibition at a fixed concentration of 10^-10^ M; FF had a significantly higher suppressive effect on IFN-γ, IL-2 and IL-17 release vs. MF (Figure 1).

**Table 1:** Nasal polyp tissue (n = 9) was incubated with FF or MF at different concentrations for 1 hour and subsequently stimulated with SEB for 24 hours (pre-challenge incubation).

Cytokine	Fluticasone furoate			p- value for max. inhibition	Mometasone furoate		
	*Max. inhibition (median (Interquartile range) in %*	*Concentr. of max. inhibition*	*Lowest concentr. with significant inhibition*		*Max. inhibition (median (Interquartile range) in %*	*Concentr. of max. inhibition*	*Lowest concentr. with significant inhibition*
**IFN-γ**	66 (56.5–77.5)	10^−8^ M	10^−10^ M	P = 0.7040	69 (62.8–78.)	10^−8^ M	10^−9^ M
**IL-2**	67.5 (52–75.6)	10^−8^ M	10^−10^ M	P = 0.7223	75 (46.5–79)	10^−8^ M	10^−9^ M
**IL-5**	81.5 (72–88)	10^−8^ M	10^−11^ M	P = 0.3606	89 (80.5–91)	10^−8^ M	10^−10^ M
**IL-17**	76 (68–82.5)	10^−9^ M	10^−10^ M	P = 0.7223	77 (65.8–79)	10^−8^ M	10^−9^ M
**TNF-α**	65.5 (46–75.5)	10^−8^ M	10^−10^ M	P = 0.2008	42 (32.5–69)	10^−8^ M	10^−9^ M

There was no significant difference in the maximal inhibition of cytokine release over 24 h; however, FF induced a comparable inhibition for all cytokines at lower molar concentrations.

### Upon challenge with SEB, FF has a stronger effect at lower concentrations compared to MF

**Figure 2:**
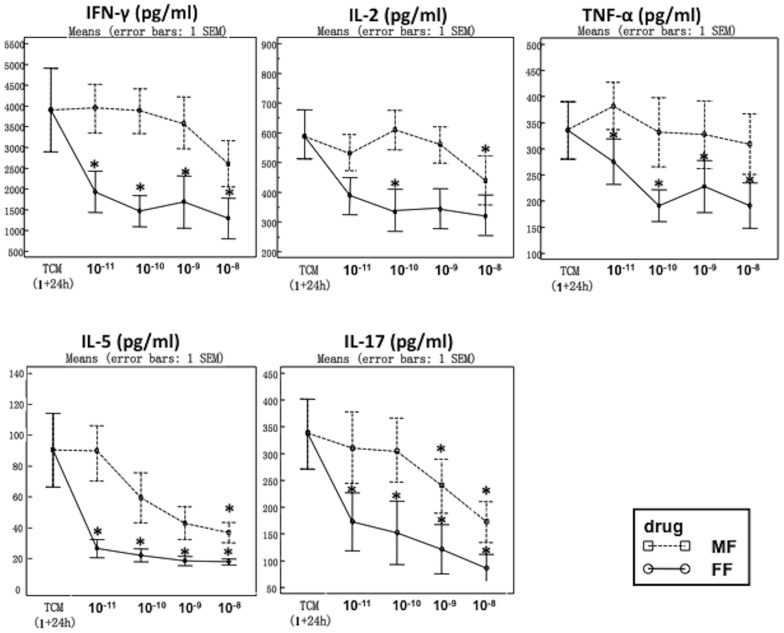
Nasal polyp tissue (n = 9) was stimulated with SEB for 1 hour before incubation with FF and MP at 10^−11^ to 10^−8^ molar concentrations for further 24 hours (post-challenge incubation). The figures show a concentration-dependent inhibition of cytokine release, reaching significance at different concentrations per drug and cytokine. Significance is indicated vs. baseline (* p<0.05). Please refer to table 2 for a comparison between drugs.

SEB stimulation resulted in a strong inflammation with the release of all cytokines, which was reduced with both drugs. Added an hour after SEB, FF showed a higher max. % inhibition on the release of IFN-γ, IL-2, IL-5, IL17, and TNF-α over 24 hours compared to MF (Table 2 and Figure 2). The maximum % inhibition was similar when drugs were added after 1, 2 or 4 h after SEB stimulation (data not provided).

**Table 2:** Nasal polyp tissue (n = 9) was stimulated with SEB for 1 hour before incubation with FF and MP at different concentrations for further 24 hours (post-challenge incubation).

Cytokine	Fluticasone furoate			p- value for max. inhibition	Mometasone furoate		
	*Max. inhibition (median (Interquartile range) in %*	*Concentr. of max. inhibition*	*Lowest concentr. with significant inhibition*		*Max. inhibition (median (Interquartile range) in %*	*Concentr. of max. inhibition*	*Lowest concentr. with significant inhibition*
**IFN-γ**	77 (71.8–78.5)	10^−8^ M	10^−11^ M	P = 0.0005	41 (33.8–56)	10^−8^ M	N
**IL-2**	64 (55–67)	10^−8^ M	10^−10^ M	P = 0.0027	33 (20–46)	10^−8^ M	N
**IL-5**	73 (67.5–87)	10^−8^ M	10^−11^ M	P = 0.0340	58 (31.8–73.3)	10^−8^ M	10^−8^ M
**IL-17**	80 (72.5–82.8)	10^−8^ M	10–11 M	P = 0.0092	58 (42–63	10–8 M	10–9 M
**TNF-α**	46 (40.8–63.8)	10^−9^ M	10–11 M	P = 0.0047	29 (12.8–38.5)	10–8 M	N

We observed a significantly better maximal inhibition for all cytokines with FF compared to MF. Furthermore, a significant inhibition was reached for all cytokines with FF, whereas MF failed to significantly suppress IFN-γ, IL-2 and TNF-α. For IL-5 and IL-17, a significant inhibition was reached with FF at one order of magnitude lower molar concentrations compared to MF.

N = no significant reduction reached at any concentration tested.

### Onset of action is significantly faster with FF vs. MF, and the suppressive effects of FF are superior to MF for IFN-γ, IL-2 and IL-17

**Figure 3:**
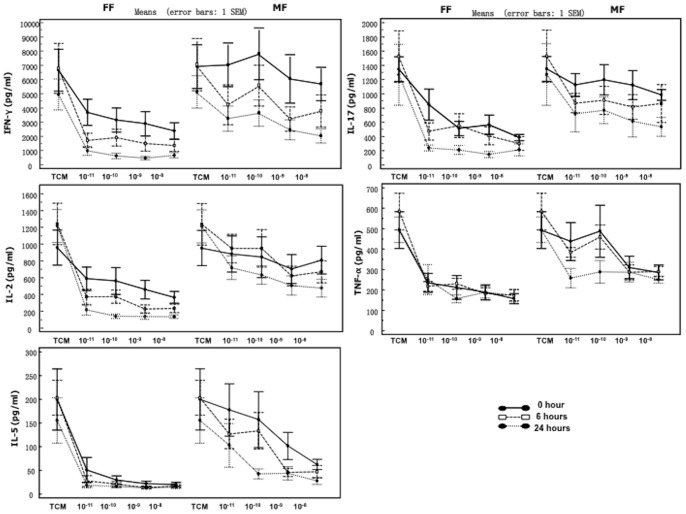
Nasal polyp tissue (n = 9) was incubated with FF and MF at different concentrations for 1 hour, then washed and SEB was added either immediately (0 hour), after 6 or 24 hours (pre-challenge incubation with time interval) Cells were then incubated for another 24 hours. When SEB stimulation was performed immediately, FF demonstrated a faster onset of action with a significantly higher maximal inhibition of all cytokines vs. MF. For most cytokines and time points, FF reaches a significant reduction at a lower concentration; however, when the time interval between pre-incubation and challenge was minimally 6 hours, the suppression capacity of MF reached the same levels as FF for IL-5 and TNF-α.

Polyp tissue fragments were incubated with FF or MF for 1 hour, then washed to remove drug not bound to the tissue, and stimulated with SEB immediately or after 6 or 24 hours. Both drugs showed a time dependent dynamic suppression of IFN-γ, IL-2, IL-17 and TNF-α release. FF showed a much faster uptake and suppressive effect after 1-hour incubation compare to MF, the max % inhibition was significantly superior for FF vs. MF (P<0.05). Especially for IL-5, 1-hour incubation of the tissue with FF resulted in an immediate maximum % inhibition, whereas an inhibitory effect in the same range needed at least 6 hours with MF (Table 3 and Figure 3). For other cytokines, the inhibitory activity of FF always remained superior to MF even when stimulated after 6 or 24 hours.

**Table 3:** Nasal polyp tissue (n = 9) was incubated with FF and MF at different concentrations for 1 hour, and then washed and SEB was added immediately (0 hour), after 6 or 24 hours (pre-challenge incubation with time interval).

Cytokine		Fluticasone furoate				Mometasone furoate		
	*time*	*Max. inhibition (median (Interquart. range) in %*	*Concentr. of max. inhibition*	*Lowest concentr. with significant inhibition*	*p-value for max. inhibition*	*Max. inhibition (median (Interquart. range) in %*	*Concentr. of max. inhibition*	*Lowest concentr. with significant inhibition*
**IFN-γ**	0 h	63 (58.3–78)	10^−8^ M	10^−11^ M	**P = 0.0054**	34 (9,5–43.5)	10^−8^ M	10^−8^ M
	6 h	86 (82.5–90)	10^−8^ M	10^−11^ M	**P = 0.007**	71 (36.8–81.3)	10^−9^ M	10^−9^ M
	24 h	94 (91.3–96)	10^−9^ M	10^−11^ M	**P = 0.0033**	70 (55.5–73.5)	10^−8^ M	10^−9^ M
**IL-2**	0 h	65 (50–73.8)	10^−8^ M	10^−11^ M	**P = 0.0092**	39 (13.5–43.8)	10^−9^ M	10^−9^ M
	6 h	90 (79.591.5)	10^−9^ M	10^−11^ M	**P = 0.0041**	74 (37.5–76.3)	10^−9^ M	10^−11^ M
	24 h	93 (87.5–95)	10^−10^ M	10^−11^ M	**P = 0.0297**	64 (60.8–86)	10^−8^ M	10^−11^ M
**IL-5**	0 h	90 (80.8–94.3)	10^−8^ M	10^−11^ M	**P = 0.0118**	51 (27.8–73.3)	10^−8^ M	10^−8^ M
	6 h	90 (81.5–97.3)	10^−9^ M	10^−11^ M	P = 0.1022	76 (63.5–90.3)	10^−9^ M	10^−9^ M
	24 h	85 (84.3–95.8)	10^−11^ M	10^−11^ M	P = 0.1599	82 (67.5–92.5)	10^−8^ M	10^−11^ M
**IL-17**	0 h	78 (72.3–85)	10^−8^ M	10^−11^ M	**P = 0.0027**	47 (28.5–70.3)	10^−8^ M	10^−8^ M
	6 h	83 (77.8–92.3)	10^−8^ M	10^−11^ M	**P = 0.038**	72 (49–79.5)	10^−9^ M	10^−11^ M
	24 h	90 (84.8–94.8)	10^−10^ M	10^−11^ M	**P = 0.0181**	70 (60.8–79.5)	10^−8^ M	10^−10^ M
**TNF-α**	0 h	70 (64–78)	10^−11^ M	10^−11^ M	**P = 0.0054**	38 (25.3–54.5)	N	N
	6 h	70 (58.3–76)	10^−11^ M	10^−11^ M	P = 0.112	63 (23–66.3)	10^−9^ M	10^−10^ M
	24 h	74 (60.5–81.8)	10^−10^ M	10^−11^ M	P = 0.2248	61 (55.3–69.5)	10^−8^ M	10^−11^ M

Cells were then incubated for another 24 hours. When SEB stimulation was performed immediately, FF demonstrated a faster onset of action with a significantly higher maximal inhibition of all cytokines vs. MF. For most cytokines and time points, FF reached a significant reduction at a lower concentration; however, when the time interval between pre-incubation and challenge was minimally 6 hours, the suppression capacity of MF reached the same levels as FF for IL-5 and TNF-α.

N =  no significant reduction reached at any concentration tested.

Stimulation with SEB after 24 hours revealed a true 24 hours effect of FF on cytokine release in the range of 74 to 94% for all cytokines, after just one hour of incubation with the compound.

## Discussion

In the current investigation we compared the effects of fluticasone furoate (FF) and mometasone furoate (MF) on the release of a pattern of Th1, Th2 and Th17 cytokines from human nasal polyp tissue. We simulated three possible real-life drug-use patterns including pre- and post-challenge exposure with FF or MF. In all experimental settings we observed stronger effects of FF compared to MF in terms of concentrations necessary to suppress cytokine release, onset of action, maximal inhibition of release and range of cytokines that could be suppressed.

A typical clinical situation would be the initiation of drug therapy after allergen exposure or onset of symptoms. This setting was mimicked by a post-challenge incubation with a concentration range of 10^−8^ to 10^−11^ M FF and MF. We observed a concentration-dependent inhibition of cytokine release, which was stronger for FF compared to MF. The maximal inhibition achieved by FF was statistically significantly higher than for MF, which did not suppress IFN-γ and TNF-α. The experimental setting can be reversed to a pre-challenge exposure resembling a prophylactic use of drugs. In this setup the corticosteroids were pre-incubated with the nasal polyp tissue fragments for one hour before SEB was supplemented. Under these conditions, the maximal cytokine inhibition was not significantly different between FF and MF, but FF consistently inhibited cytokine release at lower concentrations compared to MF. At concentrations as low as 10^-10^ M, FF showed a significantly higher suppressive effect versus IL-2, IL-17 and IFN-γ secretion compared to MF. Interestingly, when the extent of inhibitory effects in this pre- versus the post-challenge exposure setting are compared, it is striking that FF produced maximal inhibition of IL-2, IL-5, IL-17, IFN-γ and TNF-α in comparable magnitudes in both experimental setups. In contrast, the maximal inhibition of cytokine release by MF was compromised in the post-challenge incubation setting.

The third experimental setting we designed to mimic a preventive use of intranasal glucocorticoids. In a clinical context, any excess drug that is not taken up into the nasal tissue cells will be rapidly removed by mucociliary clearance within about 30 min [11]. This was mimicked by washing the tissue in our setup. Subsequently, exposure with SEB was either performed immediately after washing off excess glucocorticoids (t = 0), or after another 6 or 24 hours, respectively. Generally, FF inhibited the cytokine release significantly stronger and at earlier time points than MF. Especially the release of IL-5 and TNF-α was most effectively suppressed even by low concentrations of FF and irrespectively of the time interval between removal of the drug excess and exposure with SEB. Thus, the inhibitory activity of FF fully unfolded after only one hour incubation with the drug and was not attenuated over the evaluated time interval. The observation that FF still yielded a very potent suppression in cytokine production by SEB after 24 hours, while the tissue fragments were only exposed to the drug for one hour, is consistent with our previous results that showed a fast uptake and a high binding of FF to human nasal tissue [7] and with the report that FF was still detectable in cell nuclei 30 hours after treatment with this drug [12], [13]. Moreover, FF revealed a longer duration of action, characterized by inhibition of GM-CSF release compared to fluticasone propionate and budesonide.

However, in this pre-challenge incubation with subsequent drug removal not all cytokines were influenced to the same extent by the two corticosteroids. Specifically, the maximal inhibition of release of IL-5 and TNF-α was similar for FF and MF when the human nasal polyp tissue was challenged with SEB 6 and 24 hours after incubation with the glucocorticoids. In contrast, this was not observed for IL-2, IL-17 and IFN-γ, the release of which was significantly more inhibited by FF compared to MF over all analyzed time intervals. Furthermore, the release of IL-5 and TNF-α was already almost completely blocked by lowest concentrations of FF right after pre-incubation (t = 0), indicating a fast translocation of the drug and a fast onset of action. The observation that the influence of the glucocorticoids was distinct for certain cytokines might reflect differences in the regulation and/or mechanisms of cytokine secretion [14]. Cytokines are typically produced as response to T-cell receptor stimulations either by upregulation of their transcription or by translation of pre-existing mRNA transcripts [15].

The overall more pronounced anti-inflammatory activity of FF compared to MF is highly consistent with the higher relative receptor affinity (RRA) of FF compared to MF [6], [16].

In our experimental setting the nasal mucosal cells were in contact with the glucocorticoids typically for one hour, with the readout performed 24–48 hours later; it can be assumed that classical effects on transcription and translation of genes encoding inflammatory mediators were responsible for the observations [17]. Rapid effects or nongenomic actions also have been described within seconds to a few minutes [18]–[23].

Although an inhibition of inflammatory mediators is the prerequisite for clinical symptom management in allergic rhinitis, the required magnitude and temporal relation of cytokine suppression and the improvement of symptoms is not definite. Lately, both the FDA and the EMA issued recommendations for including the onset of action into the evaluation of drugs for allergic rhinitis [24], [25] which confer with the proposal of an EAACI workshop group [26]. Efficacy of intranasal corticosteroids can be expected after 7–8 hours of dosing, but some patients appear to benefit more rapidly, e.g. within the first two hours [2], [27]. In case of rapid improvement of clinical symptoms nongenomic glucocorticoid effects may play a role as well.

Limitations to this study include the fact that the clinical importance of these observations is unknown, and that the mechanisms underlying the observations are not identified. The study also uses whole tissue fragments and is thus not suitable to study effects on single cell components, but rather was aimed to resemble the in vivo situation in humans as much as possible.

To summarize, in the present study we demonstrated the potential of our human mucosal model and compared for the first time the activity of two modern intranasal glucocorticoids regarding inhibition of Th1, Th2 and Th17 cytokine release from human nasal polyp tissue. Thereby, we employed pre- and post-challenge incubations with FF or MF to mimic typical clinical situations of preventive use or therapy after exposure to allergens. All results revealed an uptake, receptor activation and prolonged binding of the drugs in the mucosal tissue with a rapid uptake and sustained suppression of cytokine release that was stronger for FF compared to MF. While the maximal suppressive activity was not different when FF was added before or after tissue stimulation with SEB, the cytokine suppression capacity of MF appeared to be compromised if the SEB induced cell activation preceded the drug addition. In a pre-challenge incubation with removal of excess drug concentrations MF approached inhibition of IL-5 and TNF-α comparable to FF. However, this was only observed after 6 and 24 hours while FF maximally blocked the release of these cytokines already right after pre-incubation and at concentrations up to four degrees of magnitude lower than for MF. Furthermore, there was a difference in the cytokines that were suppressed, with a wider range of T helper signatures for FF compared to MF. These data underline the high efficacy of FF in allergic and other inflammatory diseases and indicate the necessity of head-to-head comparison studies *vs*. MF and other corticosteroids in adequate clinical settings using appropriate concentrations.
